# Robot-assisted laparoscopic orchiopexy: A comparative analysis with laparoscopic orchiopexy

**DOI:** 10.3389/fruro.2023.1103915

**Published:** 2023-01-30

**Authors:** Adam J. Rensing, Abdul Qadar, Clark Higganbotham, Dominic Frimberger, Bhalaajee Meenakshi-Sundaram

**Affiliations:** University of Oklahoma Health Sciences Center (HSC), Department of Urology, Oklahoma City, OK, United States

**Keywords:** cryptorchidism, undescended, laparoscopy, robot-assisted surgery, cost comparative analysis

## Abstract

**Background:**

While undescended testes are relatively common, the nonpalpable testis remains a challenging problem. The gold standard treatment remains the laparoscopic orchiopexy. However, today robot-assisted surgery has challenged traditional laparoscopy in many areas of urology. Yet the value of this new approach remains controversial, given concerns with operative time and cost. We reviewed our contemporary results of both robot-assisted orchiopexy (RALO) and traditional laparoscopic orchiopexy (TLO). Our primary aims were to retrospectively compare success rates, and operative time. Our secondary aims were to compare costs and complications related to each method.

**Methods:**

In this study, all patients treated with laparoscopic and robot-assisted laparoscopic orchiopexy from April 2017 to January 2022 were reviewed using CPT code 54692. Exclusion criteria included bilateral concomitant orchiopexy or concomitant “major surgery,” or follow up less than 6 months. Also excluded were 1^st^ stage orchiopexies. For the purposes of comparison, 1-stage and 2^nd^ stage orchiopexies were included in the analysis. Patient demographics, surgical operative notes and documentation were all reviewed.

**Results:**

After exclusion criteria was applied, 16 and 17 remained in the laparoscopic and robot-assisted cohorts, respectively. Upon follow up, all patients in both the laparoscopic and robot-assisted cohorts were noted to have a healthy testicle palpable in the dependent portion of the scrotum. The median operative time in the TLO group was 71 minutes, compared to 101 minutes in the RALO group (p>0.0001). When comparing median hospital charges, the TLO group was lower at $38,813, compared to $46,455 in the RALO group (p = 0.0069). There was one postoperative complication in the TLO group (localized wound infection), compared to zero in the RALO group.

**Conclusions:**

The robot-assisted orchiopexy is safe and effective. However, at this time it remains more costly in terms of time and resources.

## Introduction

Undescended testes remain a common consult. In particular, the nonpalpable testis provides a unique surgical challenge. This situation requires determining the position of the testis if it is present. Diagnostic laparoscopy remains the gold standard for identifying the presence and location of a nonpalpable testis. Traditionally, laparoscopic orchiopexy has been performed in the same setting if a testis is present within the abdomen (1). As with other surgeries involving delicate structures, the traditional laparoscopic approach to orchiopexy is technically challenging. Today, with a shift in case volume from traditional laparoscopy toward robot-assisted laparoscopic surgery, conversely younger surgeons are increasingly less experienced with traditional laparoscopy, and more comfortable with the da Vinci platform both in urology, and even pediatric surgery (2–4). However, the integration of robotics into urology residency programs is not yet formalized (5).

More recently, robot-assisted surgery has begun to play a larger role in urologic surgery. Within adult urologic surgery, robot-assisted surgery has supplanted laparoscopic surgery in prostate and renal surgery (6). In the pediatric realm, robot-assisted surgery has also become increasingly common. This has been best demonstrated in the shift in technique for pyeloplasty. This shift from traditional laparoscopy toward the da Vinci™ system has been reflected in trainee experience. Yet, detractors point out that the benefits of robot -assisted urologic surgery have oftentimes been overstated or a tradeoff with unique downsides, specifically in the pediatric arena (7, 8).. With an increasing sensitivity toward surgical costs, some have questioned the value of this technology’s adoption (9, 10). As with any new surgical technology, the urologic community is learning what operative goals are best approached with robot-assistance.

At our institution, with one seasoned laparoscopic surgeon and two younger surgeons familiar with robot-assisted surgery, we have developed concomitant cohorts of traditional laparoscopic orchiopexy (TLO) and robot-assisted laparoscopic orchiopexy (RALO). More specifically, we sought to retrospectively compare success rates and operative time between the two groups. This was done retrospectively. A secondary aim was to examine comparative hospital charges and complications between these two cohorts. To our knowledge, this is the first large series examining the efficacy and safety of robot-assisted orchiopexy (RALO).

## Materials and methods

### Operative steps for laparoscopic orchiopexy

An exam under anesthesia was performed to search for a testis in the groin. If a testis remained nonpalpable, a diagnostic laparoscopy *via* a 5 mm umbilical port was performed to identify if a testis was present within the abdomen. If a viable, intrabdominal testis was noted, two additional 5 mm laparoscopic ports were placed, roughly in a straight line with the previous port. The gubernaculum is divided. The testis is delicately mobilized while preserving the testicular vessels and vas deferens with its associated peritoneal leaflet and deferential artery. At this point, the surgeon decided whether a Fowler-Stephens procedure is necessary, based upon the testis’ tension due to the gonadal vessels when attempting to reach the contralateral internal ring. If proceeding in one stage, a transcrotal port was placed medial to the inferior epigastric, and a laparoscopic grasper was used to relocate the testis in the dependent part of the scrotum.

### Operative steps for robot-assisted laparoscopic orchiopexy

An exam under anesthesia was performed to search for a testis in the groin. If a testis remained nonpalpable, a diagnostic laparoscopy *via* a 5 mm umbilical port was performed to identify if a testis was present within the abdomen. If a viable, intrabdominal testis was noted, two additional 8 mm robotic ports were placed, roughly in a straight line with the now 8 mm robotic, umbilical port, perpendicular to the internal ring of the side of interest. After docking the robot, the testis’ vessels, vas deferens, and adjacent anatomy were identified. The gubernaculum was divided and used as a handle to manipulate the testis. The posterior peritoneum was then lifted off the testicular vessels so the vessels can be mobilized as proximally as possible. The medial extent of the vas deferens was also mobilized along with a wide leaflet of associated peritoneum. At this point, the surgeon decides whether a Fowler-Stephens procedure is necessary, based upon the testis’ tension due to the gonadal vessels when attempting to reach the contralateral internal ring. If proceeding in one stage, a transcrotal port was placed medial to the inferior epigastric, and a laparoscopic grasper was used to relocate the testis in the dependent part of the scrotum. For a video representation our approach, please refer to Higganbotham et al. (11).

### Study design

After obtaining institutional IRB approval at our institution, all consecutive patients treated at our tertiary, children’s hospital for a viable intrabdominal testis *via* CPT code 54692 were collected from April 2017 to January 2022. This code encompasses all laparoscopic (TLO) and robot-assisted laparoscopic orchiopexies (RALO). We included all 1-stage or 2^nd^/final stage orchiopexies for purposes of this study. One surgeon at our institution only performed TLO, and two others only performed RALO on all intrabdominal testes. We collected general demographic data, operative time, any and all postoperative complications, and any/all hospital charges associated with each patient’s orchiopexy. Exclusion criteria included bilateral concomitant orchiopexy or concomitant “major surgery.” We excluded all cases with less than 6 months of confirmed follow-up. Also excluded were 1^st^ stage orchiopexies. We defined “major surgery” as requiring >30 minutes and/or another surgical service. Examples included gastrostomy tube placement or contralateral inguinal hernia repair. Follow up was documented from the electronic medical record. For those not seen for an extended period of time, a phone call was attempted to family to discuss the patient.

Operative times were calculated by accessing nursing and anesthesia records regarding surgery start and end times. Operative charges were collected from the hospital. Charges were subdivided by equipment charges, anesthesia charges, and facility/operating room charges. The latter two were positively correlated with operative time. Equipment charges included the cost of disposable equipment, one tenth (1/10^th^) of the cost for any used robotic instrument (given their use). Importantly the costs of ownership of the da Vinci system, including acquiring the system, depreciation costs, and maintenance costs, were excluded, but these costs were also not included in the charges for any particular case.

### Study analysis

For analysis, the TLO and RALO cohorts were compared using a contemporary version of Microsoft Excel. Given the small sample sizes, a two-tailed Mann-Whitney U test was used to compare the TLO and RALO cohorts in all continuous variables and assess for statistical significance.

## Results

From April 2017 to January 2022, a total of 29 traditional laparoscopic orchiopexies (TLO), and 28 robot-assisted orchiopexies (RALO) were collected. Of the 29 TLOs performed, 5 surgeries were excluded, two for being first stage procedures, two for being bilateral procedures, one for complex concomitant procedure. An additional 8 cases were excluded due to inadequate follow up. This left 16 TLO cases to be reviewed. Of the 28 RALOs performed, two were excluded for being first stage procedure, one for being bilateral and two for concomitant “major” procedures, and one for orchiectomy due to vasal injury during second stage FS. An additional five were excluded due to inadequate follow up. This left 17 robot-assisted orchiopexies to be analyzed.

Both groups were roughly similar in age, race, insurance status and complications (**Table 1**). The follow up time was longer in the TLO group, given that the RALO technique was begun later (2019). Upon the last follow up visit, all patients in each cohort were found to have a palpably normal testicle within the scrotum. The Fowler-Stephens procedure was utilized less frequently in the traditional laparoscopic group (2 procedures, 13%) as compared to the robot-assisted group (10 procedures, 59%). No testis was noted to be atrophic upon follow up upon physical exam. The one postoperative complication was a patient in the TLO group who developed a wound infection in the scrotum requiring oral antibiotics. This patient was excluded due to inadequate postoperative follow up. One of the excluded RALO patients mentioned above had a long-looping vas deferens that was injured during incision of the gubernaculum.

**Table 1:** Demographics of Patients.

Demographic	Laparoscopic	Robotic
Number of testicular units	16	17
Median Age (IQR)	1.3 years (0.95-1.8)	1.15 years (0.83-1.7)
Medicaid (Percentage)	10 (63%)	12 (71%)
Race (Percentage)	11 White (69%)	11 White (65%)
	5 Other (31 %)	6 Other (36%)
Fowlers-Stephens Utilization	2 (13 %)	10 (59%)
Median Follow Up (IQR)	1228 days (1104-1525)	790 days (400-994)
Complications	0	0

**Figure 1:**
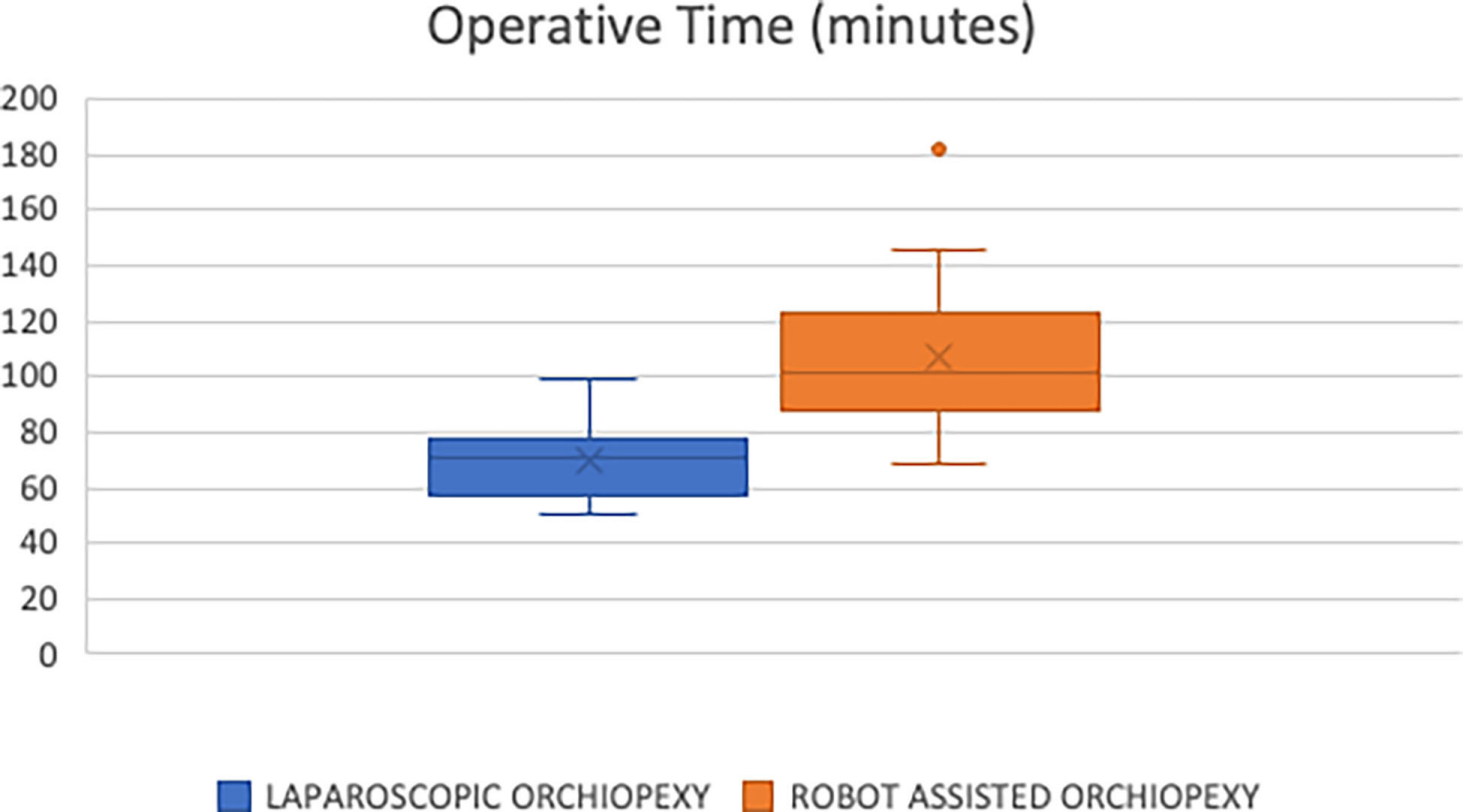
Operative time (minutes). “x”= mean of each cohort. p < 0.0001.

We compared the two groups in regard to operating room time. The median and mean times for the TLO group were 71 and 70 minutes, respectively (**Figure 1**). This was less than the RALO group with a median and mean time of 101 and 107 minutes, respectively. We found this difference to be statically significant (p<0.0001).

**Figure 2:**
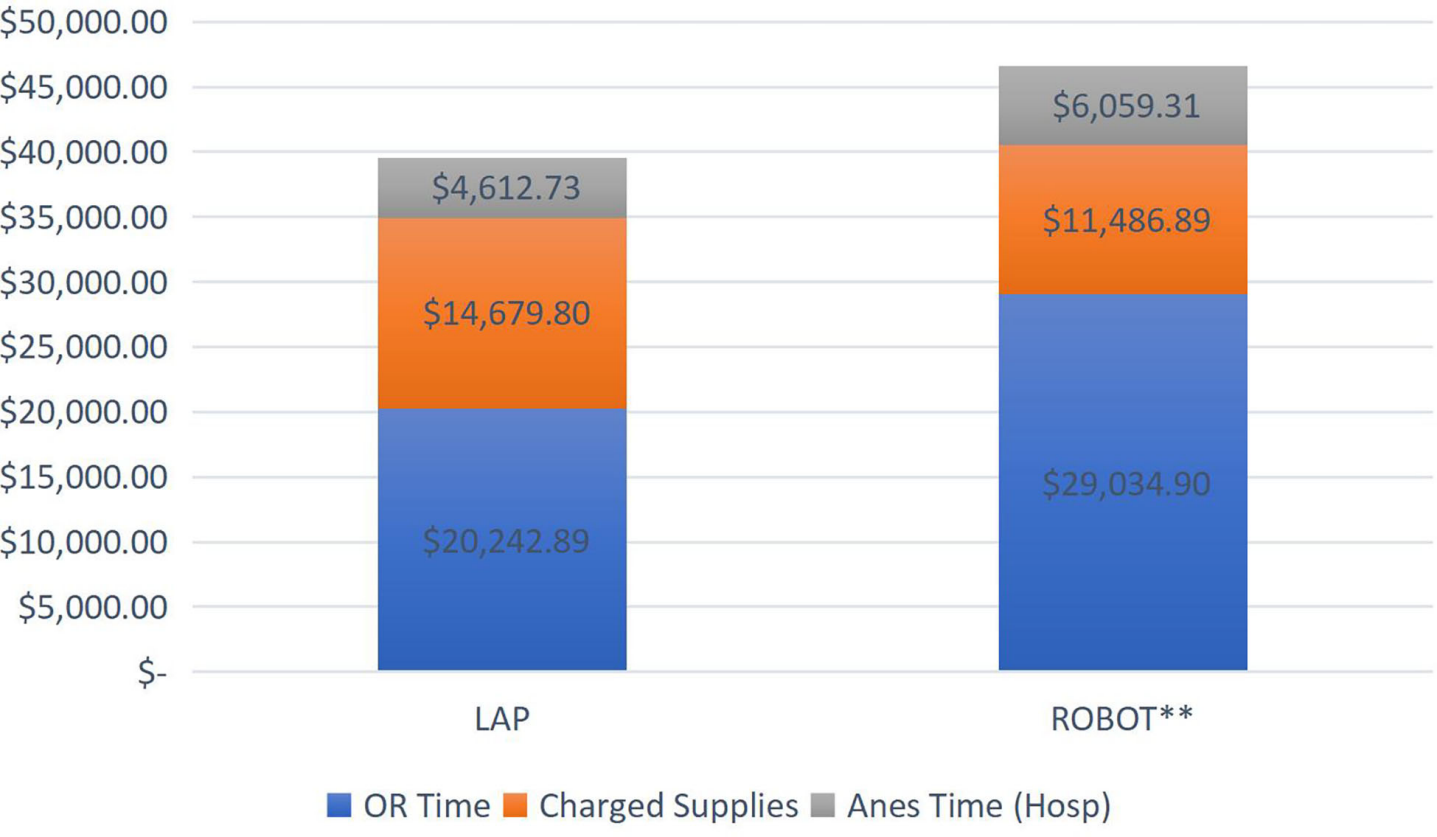
*Of note, this excludes the upfront costs sustained by the hospital to purchase the da Vinci system since these costs are not passed on to the patient/insurer. ** For the robot assisted cases, p=0.014.

In regard to hospital charges, we also saw relatively higher charges in the RALO group. For the TLO group, the median and mean total charges were $38,8213 and $39,535, respectively (**Figure 2**). For the RALO group, the median and mean total cost were $46,455 and $46,581 respectively. This difference was statistically significant (p = 0.014). In both groups, general operating room charges were the predominant charge, contributing to an overall higher charge in the RALO group. Upfront capital costs for the da Vinci system ™ are not passed on to the patient/insurer, and so no included in this analysis. Interestingly, instrument charges were lower in the RALO group, but this was a smaller component overall, and did not make up for the significantly higher operating room charges in that group.

Given that RALO was new to both our surgeons and operating room staff, we analyzed how operating room time and charges changed over time in the included procedures. We found that there was some variability in both time and cost of the RALO procedure, with no clear trend downward.

## Discussion

Robot-assisted laparoscopic orchiopexy (RALO) is a new and alternative way to approach the intrabdominal testis. It appears to be safe with a low complication rate. However, operating room time and charges seem to be significantly more with this approach, as compared to the traditional laparoscopic orchiopexy (TLO).

We found that costs were in general higher in the RALO cohort. As mentioned above, this was due to the longer time required in these cases, leading to higher OR and anesthesia costs. However, the smaller “charged supplies” category was lower in the RALO group. This seemed counterintuitive. However, at our institution, all robotic ports were reusable. In addition, any robotic instruments were listed as 1/10^th^ of charge due to 10 “lives” listed for each instrument. This differed from laparoscopic instruments, which were listed as exclusively disposable. Hanson et al. sought to reduce the cost of pediatric laparoscopic procedures by disposing of the working ports entirely by using stab incisions for access (12). This approach saved an average of $277 per case with no reported intra or postoperative complications with this approach. This technique in the TLO cohort, could further reduce the cost.

In 2004, Lorenzo et al. published a fascinating review of the cost effectiveness of laparoscopic exploration for the unilateral nonpalpable testis (13). After accounting for the relative frequency of a host of varied findings, they found that if operative time of laparoscopic exploration did not exceed 19 minute and the cost of 147 US dollars, initial laparoscopic exploration is cost effective. Powell et al. evaluated their institution’s results and costs when comparing standard laparoscopic orchiopexy to the single-stage or two-stage FS orchiopexy (14). They found that by not ligating the vessels, they achieved similar, if not better results, while reducing costs and complications.

O’Kelly et al. published a descriptive review on the cost of robotic-assisted surgery in pediatric urology (15). In their paper, the authors survey the mixed results. Despite garnering greater interest due to the more favorable learning curve, pediatric robot-assisted surgery has not been proven to provide superior results, and the costs have been prohibitive for many centers. They quote Casella et al., describing a comparison between robotic-assisted laparoscopic pyeloplasty and traditional, laparoscopic pyeloplasty (16). This study demonstrated similar costs, and consistently shorter operative times in the robotic cohort.

Most recently, Shumaker and Neheman published their series of Robot-assisted Modified One-Stage Orchiopexy (17). Here, they describe their technique, which was very similar to ours, however, all patients had a single stage orchiopexy. In addition, they described an operative time of 97 minutes (IQR 77.5-109.5). This was very similar to our RALO operative time of 101 minutes (IQR 90 – 121). Our cohort was more varied, with 5/17 (29.4%) being 2^nd^ stage Fowler Stephens orchiopexies, and 7/17 (41.2%) being a single stage orchiopexy without Fowler Stephens. Only 5/17 (29.4%) were single stage FS, robot-assisted orchiopexies. 1^st^ Stage orchiopexies were excluded from our analysis.

Our study has limitations. The distance between the internal ring and the testis was not consistently defined in reviewed operative reports. In addition, both TLO and RALO cohorts were varied in the approach. Fowler Stephens was not used consistently, and some patients were staged. With that said, both cohorts were relatively small, making durable comparisons difficult. As stated above, all TLOs were performed by an experienced pediatric, laparoscopic surgeon, while RALO was a new approach. This may have contributed to longer operative time due to surgeons and OR staff adjusting to a new procedure using the robot. The magnitude of this bias is difficult to assess. Lastly, other institutions may process their operative charges differently.

However, we feel this study provides meaningful data as to the feasibility of the robot-assisted orchiopexy. There has not been a cohort of this size reporting RALO results, especially compared to traditional laparoscopy. Although this approach was more time intensive and costly, one could argue that with larger numbers and experience, the gap could narrow with traditional laparoscopy, much like other urologic reconstructive procedures (3). Further, with skill sets shifting, younger pediatric urologists are inevitably applying the robotic skill set to urologic problems, pediatric and adult alike. Further study remains necessary to see where the robotic platform is superior or inferior to the “gold standard” approach.

## Conclusions

Robot-assisted orchiopexy (RALO) is a safe and effective approach to the intrabdominal, viable testis. We present the first large series with this new approach. Further prospective studies *via* multiple institutions are required comparing RALO and TLO to more objectively assess the value of the RALO in the pediatric urology armamentarium.

## Data availability statement

The datasets presented in this article are not readily available because they contain potentially identifiable patient information. Requests to access the datasets should be directed to Adam-Rensing@ouhsc.edu.

## Ethics statement

The studies involving human participants were reviewed and approved by The University of Oklahoma Institutional Review Board H.R.P.P. Written informed consent from the participants’ legal guardian/next of kin was not required to participate in this study in accordance with the national legislation and the institutional requirements. Written informed consent was obtained from the minor(s)’ legal guardian/next of kin for the publication of any potentially identifiable images or data included in this article.

## Author contributions

All authors contributed to the study conception and design. Material preparation, data collection and analysis were performed by AR, AQ, and BM-S. The first draft of the manuscript was written by AR and all authors commented on previous versions of the manuscript. All authors read and approved the final manuscript.
